# Clinical Effectiveness of Salvia officinalis in Periodontitis: A Split-Mouth Randomized Controlled Trial

**DOI:** 10.7759/cureus.58582

**Published:** 2024-04-19

**Authors:** Ismael W Aljuboori, Maha S Mahmood, Sarah A Al-Rihaymee

**Affiliations:** 1 Department of Periodontics, College of Dentistry, Ashur University, Baghdad, IRQ; 2 Department of Periodontics, College of Dentistry, University of Baghdad, Baghdad, IRQ; 3 Department of Periodontics, College of Dentistry, University of Babylon, Hillah, IRQ

**Keywords:** antibiotics, herbal extraction, root surface debridement, periodontitis, salvia officinalis

## Abstract

Background

Periodontitis is a chronic inflammatory condition that destroys the tissues supporting the teeth. Conventional nonsurgical treatments, such as mechanical scaling and root surface debridement (RSD), often require adjunct therapies to enhance outcomes due to their limited efficacy in completely eradicating pathogenic microorganisms. Given the adverse effects of standard adjunctive therapies, including antibiotics and nonsteroidal anti-inflammatory drugs, *Salvia officinalis *(sage) presents a promising herbal alternative due to its anti-inflammatory and antimicrobial properties. This study aims to assess the local application of Salvia officinalis gel as an adjunctive to scaling and RSD to manage periodontitis.

Methods

We conducted a randomized, controlled split-mouth clinical trial involving 14 systemically healthy periodontitis patients. We included patients with at least 20 natural teeth, a probing pocket depth (PPD) ≥5 mm, and attachment loss ≥4 mm at a minimum of five sites. Primary outcomes measured were bleeding on probing (BOP), PPD, and relative attachment level (RAL). The *Salvia officinalis *gel was applied to designated test sites post-RSD, while control sites received no adjunctive treatment. Clinical parameters were recorded at baseline and a one-month follow-up visit.

Results

The cohort consisted of 10 men and four women, with a mean age of 37.1 ± 5.46 years. At the follow-up visit, the test group demonstrated a significant reduction in mean BOP (P = 0.0004), whereas the control group showed no significant change (P ≥ 0.05). Both groups experienced significant decreases in mean PPD and RAL from baseline to follow-up, with the test group showing greater improvements.

Conclusions

*Salvia officinalis* gel, used as an adjunct to scaling and RSD, significantly improves clinical periodontal parameters in patients with periodontitis. Its anti-inflammatory properties likely underpin the observed benefits, offering an effective and safe alternative to traditional chemical pharmaceuticals. Further research is needed to explore the long-term effects and mechanisms underlying the therapeutic benefits of *Salvia officinalis* in periodontal treatment.

## Introduction

Periodontitis is a longstanding inflammatory disease marked by the breakdown of tissues that support the teeth, characterized by progressive destruction of the dentoalveolar attachment system [1]. Successful treatment outcomes from nonsurgical therapy focus on eliminating bacterial infections to prevent disease progression, reduce soft tissue inflammation, and reattach periodontal tissues to the originally diseased root surface [2]. The most common nonsurgical periodontal therapy is mechanical scaling and root surface debridement (RSD) using hand tools or ultrasonic scalers. However, RSD procedures often fail to completely remove pathogenic microorganisms and their byproducts from periodontal pockets [3]. Therefore, adjuncts to RSD, such as antimicrobial agents [4,5] and laser therapy [6,7], have been employed to improve treatment outcomes. Nonetheless, the adverse effects of antibiotics and nonsteroidal anti-inflammatory drugs raise concerns about antimicrobial resistance and systemic alterations. Phytochemicals present a viable alternative with minimal adverse effects [8].

Herbal remedies, rich in antibacterial, antioxidant, antiseptic, anti-inflammatory, and anti-collagenase properties, have been used for centuries to treat various illnesses, including periodontitis [9-13]. *Salvia officinalis *Linnaeus (sage), belonging to the Lamiaceae family, serves multiple purposes, including culinary, medicinal, and commercial uses, due to its remarkable antioxidant and anti-inflammatory properties [14]. Mendes et al. demonstrated the antimicrobial and non-cytotoxic effects of varying concentrations of glycolic extract from *S. officinalis* [15]. This study aims to evaluate the anti-inflammatory effects of *Salvia officinalis *gel when used with scaling and RSD in treating periodontitis.

## Materials and methods

Study population

We conducted a randomized, controlled split-mouth clinical trial involving 14 systemically healthy periodontitis patients from the Department of Periodontics, College of Dentistry, Ashur University. The Research Ethics Committee of the College of Dentistry, Ashur University approved the trial, adhering to the Declaration of Helsinki principles (Approval Number 0164-CD). Inclusion criteria were patients with periodontitis, having at least 20 natural teeth, with probing pocket depth (PPD) ≥5 mm, and attachment loss ≥4 mm at a minimum of five tooth sites. We excluded pregnant or lactating patients, smokers, and those with systemic diseases affecting periodontal disease progression or requiring antibiotic use. Patients who had taken anti-inflammatory medications or antibiotics within the last three months were also excluded. Primary outcomes included clinical periodontal parameters: bleeding on probing (BOP), PPD, and relative attachment level (RAL). All patients provided informed consent after a detailed discussion about the therapy process.

Plant material

We sourced *Salvia officinalis* subshrubs from Jordan, drying the separated leaves at ambient temperatures of 20°C to 25°C, monitored with a thermometer. This non-forced drying method preserves heat-labile compounds [16].

Herbal extraction and test for flavonoids

The extraction occurred at the Ibn Al-Bitar Research Center, Federal Commission for Industrial Development, Ministry of Industry and Minerals. We used an herbal medicine disintegrator (FW177, Hangzhou Chincan Trading Co., Hangzhou, China) to grind the dried leaves. Then, we performed maceration extraction with 70% ethanol, mixing the plant material and solvent in a 1:10 ratio for optimal efficacy [17]. We macerated a 100-gram portion of plant material in one liter of 70% ethanol solution for five hours, using a refrigerated incubator shaker (SL-600R, Jeio TecH Lab Companion, Daejeon, Korea) at 45°C to expedite the process. We filtered the extract twice, first through gauze pads into a conical flask, then through filter paper in a Buchner funnel connected to a vacuum pump, ensuring a pure extract. Finally, we dehydrated the extract with a Mini spray dryer B-290 (Buchi, Flawil, Switzerland), maintaining inlet temperatures between 48°C to 50°C and outlet temperatures below 50°C, within the 42°C to 44°C range, to ensure optimal drying [18]. Mixing 1 ml of herbal extract with an equal volume of 5% potassium hydroxide in a test tube formed a dark yellow precipitate, indicating the presence of flavonoid compounds [19-21].

Gel preparation

Initially, we dissolved one gram of Carbopol 940 in 25 ml of distilled water. We added 0.4 grams of *Salvia officinalis* alcoholic extract to a 10-ml mixture comprising 5 ml of absolute ethanol, 3 ml of propylene glycol, and 2 ml of distilled water, achieving a potent 4% concentration of active ingredients. We mixed 10 ml of this extract with 10 ml of gel and adjusted the pH to above 7 using triethanolamine, resulting in a final product with a 2% concentration of both the gelling agent and *Salvia officinalis* extract. After allowing it to set for 24 hours, we transferred the gel into syringes for easy application.

Oral hygiene instructions and conventional periodontal therapy

We conducted a thorough examination during the initial visit and recorded the patient's medical and dental history. We scaled the entire dentition using an EMS Piezon® Mini-Master ultrasonic scaler (Electro Medical Systems S.A., Nyon, Switzerland). We then took an impression of the dentition to construct an acrylic stent. Each patient received personalized oral hygiene instructions, including the proper use of floss, interproximal brushes, and a soft manual toothbrush tailored to their individual needs.

Intervention

One week after the initial visit, we randomly selected two non-adjacent periodontal pocket sites in separate quadrants as either the test or control site, ensuring both exhibited positive bleeding on probing. A calibrated examiner recorded clinical periodontal parameters such as BOP, PPD, and RAL using a UNC 15 periodontal probe and an acrylic stent, setting these as baseline data.

We performed RSD at both sites using Grassy curettes and concluded the procedure once the root surfaces were confirmed to be smooth through detection with an explorer. For the test site, we applied *Salvia officinalis* gel post-RSD. We isolated the site with cotton rolls to prevent saliva contamination, then dispensed the gel into the pocket from base to marginal gingiva using a disposable syringe with a bent, blunted 25-gauge needle for ease of application.

Instructions for postoperative care

After treatment, we recommended that patients avoid brushing their teeth for the first 24 hours, suggesting they resume brushing the following day. We did not recommend using mouthwash or any prescriptions after the treatment. We scheduled a follow-up visit one month post-intervention to record BOP, PPD, and RAL.

Statistical analysis

We conducted data analysis on SPSS Statistics for Windows, Version 27.0 (IBM Corp., Armonk, USA), presenting the results as mean and standard deviation. We employed an independent t-test for comparisons between groups and a paired t-test for comparisons within groups. We considered a P-value less than 0.05 to be statistically significant.

## Results

**Figure 1:**
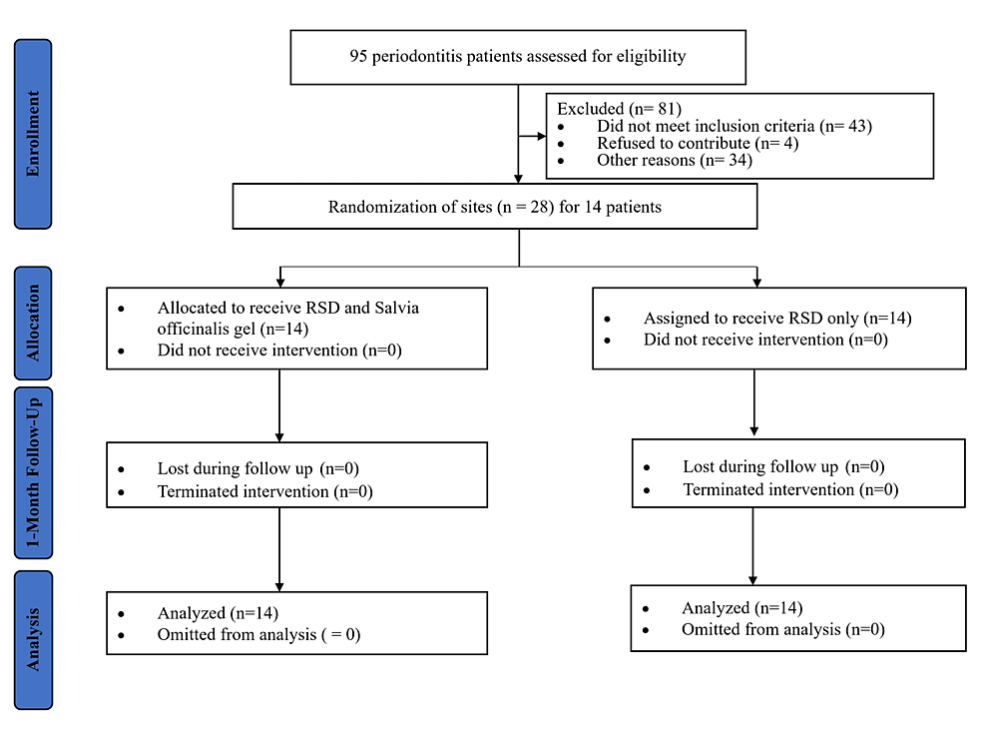
CONSORT 2010 flowchart Abbreviations: CONSORT, Consolidated Standards of Reporting Trials; RSD, root surface debridement.

Our study included a total of 14 systemically healthy patients diagnosed with periodontitis, consisting of 10 men and four women, with an average age of 37.1 ± 5.46 years (Figure 1). We found no significant differences in the baseline measures of BOP, PPD, and RAL between the study groups before the intervention (P ≥ 0.05). However, after the intervention, the test group showed a significant reduction in the mean BOP at the follow-up visit compared to the baseline (P = 0.0004), while the control group did not show a significant change in mean BOP (P ≥ 0.05). Both groups experienced significant decreases in the mean values of PPD and RAL at the follow-up visit compared to baseline. Notably, the test group exhibited greater improvements in PPD and RAL than the control group, as detailed in Table 1.

**Table 1:** Comparison between certain clinical periodontal parameters at baseline and one-month follow-up *Intergroup comparison **Intragroup comparison Abbreviations: BOP, bleeding on probing; PPD, probing pocket depth; RAL, relative attachment level; NA, not applicable.

Clinical Parameters	Study Groups	Baseline Mean ± SD	One-Month Follow-Up Mean ± SD	P-Value**
BOP (%)	Test	100 ± 0 %	56.5 ± 50.6 %	0.0004
	Control	100 ± 0 %	86.9 ± 34.4 %	0.08
	p*	1	0.02	NA
PPD (mm)	Test	5.85 ± 0.84 mm	4.39 ± 0.94 mm	0.001
	Control	5.35 ± 0.5 mm	4.6 ± 0.6 mm	0.001
	p*	0.44	0.04	NA
RAL (mm)	Test	8.5 ± 1.1 mm	7.1 ± 1.44 mm	0.001
	Control	8.39 ± 0.83 mm	7.64 ± 1.0 mm	0.001
	p*	0.77	0.03	NA

## Discussion

In recent years, there has been a surge in interest in herbal medicines, among which *Salvia officinalis*, commonly known as sage, has garnered attention in traditional medicine for its anti-inflammatory properties and diverse medicinal uses. The European Pharmacopoeia has recognized *Salvia officinalis* leaf extract as a beneficial oral solution for reducing swelling and discomfort in inflamed gingival tissues [22]. This trial aimed to evaluate the anti-inflammatory effects of *Salvia officinalis* gel when used alongside scaling and RSD in treating periodontitis.

Our findings align with previous research [15,17,18,23] that highlights the capacity of *Salvia officinalis* to modulate inflammatory responses, a critical factor in the progression and tissue destruction associated with periodontal disease. We observed a significant decrease in BOP in the test group at the follow-up visit, whereas the control group showed no statistically significant change. Although the direct relationship between BOP and *Salvia officinalis* has yet to be explored, the herb's anti-inflammatory qualities likely contributed to the significant reduction seen in the test group.

Furthermore, both groups showed a reduction in PPD at the follow-up visit compared to baseline, with the test group demonstrating a greater reduction. This finding contrasts with the study by Pistorius et al., which did not observe a significant reduction in PPD in either group [24]. The inclusion of the RSD procedure in our study, which was absent in Pistorius et al.'s research, may explain our differing results.

Additionally, we noted a significant improvement in RAL in both groups at the follow-up visit, with the test group exhibiting a more substantial gain. This improvement can be attributed to the anti-inflammatory effects of *Salvia officinalis*, facilitating faster wound healing, greater attachment gain, and further reduction in PPD, consistent with several studies [25].

This study has several limitations that warrant consideration. The small sample size and the split-mouth design may limit the generalizability of the findings to the broader population with periodontitis. The study's duration was relatively short, which may not adequately capture the long-term effects and potential benefits or risks associated with using *Salvia officinalis* gel in periodontal treatment. Additionally, the absence of a placebo control for the *Salvia officinalis* gel application might introduce bias in the results, as patient and clinician expectations could influence the outcomes. The study did not assess the potential systemic effects of *Salvia officinalis*, which could provide a more comprehensive understanding of its therapeutic profile. Future research with larger sample sizes, longer follow-up periods, and the inclusion of a placebo control would help to validate and expand upon the findings of this study.

## Conclusions

The significant improvement in BOP in the test group underscores the anti-inflammatory properties of *Salvia officinalis*. Using *Salvia officinalis *gel as an adjunct to scaling and RSD significantly enhances clinical periodontal parameters. These results suggest that *Salvia officinalis*, due to its phytochemical properties, offers an effective and safe alternative to chemical pharmaceuticals in managing and treating periodontitis.
